# Screening of Reference Genes under Biotic Stress and Hormone Treatment of Mung Bean (*Vigna radiata*) by Quantitative Real-Time PCR

**DOI:** 10.3390/genes14091739

**Published:** 2023-08-30

**Authors:** Yanyan Zhou, Huan Liu, Ting Wu, Yu Zheng, Ruimin Wang, Dong Xue, Qiang Yan, Xingxing Yuan, Xin Chen

**Affiliations:** 1 College of Life Sciences, Nanjing Agricultural University, Nanjing 210095, China2022816124@stu.njau.edu.cn (H.L.);; 2 Institute of Industrial Crops, Jiangsu Academy of Agricultural Sciences/Jiangsu Key Laboratory for Horticultural Crop Genetic Improvement, Nanjing 210014, China; 3 School of Life Sciences, Jiangsu University, Zhenjiang 212013, China

**Keywords:** hormone treatment, mung bean, reference genes, stability evaluation, soil-borne pathogens

## Abstract

Mung bean (*Vigna radiata*) production has been greatly threatened by numerous diseases. Infection with these pathogens causes extensive changes in gene expression and the activation of hormone signal transduction. Quantitative real-time PCR (qRT-PCR) is the most common technique used for gene expression validation. Screening proper reference genes for mung bean under pathogen infection and hormone treatment is a prerequisite for ensuring the accuracy of qRT-PCR data in mung bean disease-resistance research. In this study, six candidate reference genes (*Cons4*, *ACT*, *TUA*, *TUB*, *GAPDH*, and *EF1α*) were selected to evaluate the expression stability under four soil-borne disease pathogens (*Pythium myriotylum*, *Pythium aphanidermatum*, *Fusarium oxysporum*, and *Rhizoctonia solani*) and five hormone treatments (SA, MeJA, ETH, ABA, and GA_3_). In the samples from different treatments, the Ct value distribution of the six candidate reference genes was different. Under the condition of hormone treatment, the Ct value ranged from a minimum of 17.87 for *EF1α* to a maximum of 29.63 for *GAPDH*. Under the condition of pathogen infection, the Ct value ranged from a minimum of 19.43 for *EF1α* to a maximum of 31.82 for *GAPDH*. After primer specificity analysis, it was found that *GAPDH* was not specific, so the five reference genes *Cons4*, *ACT*, *TUA*, *TUB,* and *EF1α* were used in subsequent experiments. The software products GeNorm, NormFinder, BestKeeper and RefFinder were used for qRT-PCR data analysis. In general, the best candidates reference genes were: *TUA* for SA, ABA, GA3, and *Pythium myriotylum* treatment; *TUB* for ETH treatment; *ACT* for MeJA and *Fusarium oxysporum* treatment; and *EF1α* for *Pythium aphanidermatum* and *Rhizoctonia solani* treatment. The most stably expressed genes in all samples were *TUA*, while *Cons4* was the least stable reference gene. Finally, the reliability of the reference gene was further validated by analysis of the expression profiles of four mung bean genes (*Vradi0146s00260*, *Vradi0158s00480*, *Vradi07g23860*, and *Vradi11g03350*) selected from transcriptome data. Our results provide more accurate information for the normalization of qRT-PCR data in mung bean response to pathogen interaction.

## 1. Introduction

Mung bean is an important industrial crop belonging to the *Vigna* genus and is considered a medicine–food homology crop, making it one of the most significant commercial products in Asia [1,2]. Mung bean seeds are a rich source of vitamins, proteins and trace minerals, making them highly nutritious and therapeutically valuable. During production in the field, numerous diseases influence mung bean growth and development, leading to yield loss and quality degradation. Among these, soil-borne diseases caused by *P myriotylum*, *P aphanidermatum*, *F oxysporum* and *R solani* usually affect the entire developmental stage. For instance, when mung bean root rot develops, it causes the aboveground part of the plant to become dwarfed, withered, and yellow; the root starts to discolor from the tip of the root and become water-soaked; and the lower part of the main root will first appear brown, turning to russet in severe cases. The lower part of the principal root completely rots, resulting in the death of seedlings and posing a quantitative and qualitative threat to the mung bean crop [3,4,5,6]. *P. myriotylum* causes pre-emergence rot and root rot of *Phaseolus vulgaris* seedlings [3]. Turmeric rhizome rot is mainly caused by *P. aphanidermatum*, which is considered to be the principal disease that hinders the production of turmeric, resulting in a reduction in turmeric production of between 5% and 30% [7,8]. *R. solani* is one of the crucial soil-borne necrotrophic phytopathogens causing disease in staple crops, such as rice sheath blight disease, potato black scurf disease, soybean rhizoctonia foliar blight disease, and corn sheath blight [9,10,11]. *F. oxysporum* induces vascular wilt in tomato, resulting in accelerated wilting of tomato plants and 10–80% yield loss. It also causes wilt in chickpea plants, which can result in 10–100% yield loss [12,13]. In response to pathogen infection, almost the entire plant genome (97% to 99%) undergoes transcriptional modulation [14]. To understand plant–pathogen interactions, the gene expression assay is an excellent method to uncover candidate genes for the signaling and metabolic pathways.

The quantitative real-time polymerase chain reaction (qRT-PCR) method is a highly sensitive tool for detecting the expression of specific genes compared to traditional PCR [15,16,17]. There are two approaches to qRT-PCR—absolute quantification and relative quantification [18,19]. Relative quantification is the most frequently utilized approach to determine gene expression levels and differences. Nevertheless, several limitations impact the experimental outcomes, such as the RNA quality and concentration, reverse-transcription cDNA concentration, and amplification efficiency [20,21,22]. Relative quantification necessitates the use of internal reference genes to rectify the data when interpreting the results, thereby improving the accuracy of the quantitative results. The expression levels of an ideal reference gene should show little variation across different tissues, developmental stages, and various stresses [23]. Therefore, the selection of appropriate reference genes is a critical prerequisite for the accurate analysis of qRT-PCR results.

In botany, the traditionally used reference genes are usually involved in cellular biochemical pathways or important components of the cytoskeleton, such as *Actin (ACT)*, *Elongation factor-1a (EF1α)*, *α-Tubulin (TUA)*, *18S ribosomal RNA (18SrRNA)*, *β-Tubulin (TUB)*, and *Glyceraldehyde-3-phosphate dehydrogenase (GAPDH)* [24,25,26,27]. However, several studies indicate that the classic reference genes show unstable expression in many species, especially under different treatment conditions [28,29,30]. For instance, in soybean, *GmEF1α* and *GmEF1β* were most stable in roots infected with *Meloidogyne incognita*, while *GmCYP2* and *GmELF1α* were the most stable genes in leaves infested with *Anticarsia gemmatalis* [31]. To select appropriate reference genes for qRT-PCR analysis, it is important to consider their expression stability across different stress conditions. To our knowledge, no studies have evaluated the expression stability of reference genes under biological stress and hormone treatment in mung bean.

In this study, six candidate reference genes—*ACT*, *TUA*, *TUB*, *GAPDH*, *EF1α,* and *Cons4*—were selected based on the mung bean genome and related literature reports. The expression stabilities of these six candidate reference genes’ responses to infection by four phytopathogens and five hormone treatments were systematically analyzed by qRT-PCR. The gene stability analysis software GeNorm [32], NormFinder [33], BestKeeper [34] and RefFinder [35] were used to evaluate the stability of the candidate reference genes. Finally, four genes were selected according to the transcriptome data to verify the selected candidate reference genes.

## 2. Materials and Methods

### 2.1. Plant Materials and Treatments

Mung bean seeds “sulv 1” were planted in plastic pots (7 cm × 7 cm) filled with mixotrophism substrate (vermiculite/nutrient soil = 1:1). The pots were placed in a greenhouse (25 °C, 16 h light/8 h dark). Selected fourteen-day-old plants were used as subsequent experimental materials. The plant roots were immersed in mycelial liquid (1 L/dish) under soil-borne disease pathogen treatment (*P. myriotylum*, *P. aphanidermatum*, *F. oxysporum*, or *R. solani*). For hormone treatment (100 μM SA, 50 μM MeJA, 1 mM ETH, 50 μM ABA, or 50 μM GA_3_), the plant roots were immersed in different concentrations of hormone solutions. After 30 min of treatment, plant roots taken out and cultured in water. Samples were collected at 0, 12, 24, and 48 h after the respective treatments. Each time point was completed with three replicates, each comprising three plant roots, and stored at −80 °C.

### 2.2. RNA Extraction and cDNA Synthesis

An RNA extraction kit (Vazyme, Nanjing, China) was used for total RNA extraction following the manufacturer’s instructions. The RNA concentration was measured using a MICRO Spectrophotometer-k5800 (KAIAO, Beijing, China). The integrity of the RNA was verified by 1% agarose gel electrophoresis. Based on the recommendation of the HiScript III 1st Strand cDNA Synthesis Kit (Vazyme, Nanjing, China), 1 μg total RNA was used for cDNA synthesis. The total system volume was 20 µL.

### 2.3. Primer Design

According to the mung bean genome and related literature reports [36], six candidate reference genes were screened: *Cons4*, *ACT*, *TUA*, *TUB*, *GAPDH* and *EF1α* (Table 1). qRT-PCR primers were designed using Primer 5.0.

### 2.4. PCR and Quantitative Real-Time PCR Analysis

To detect the specificity of the primers, 1% agarose gel electrophoresis was performed. The reaction mixture consisted of 10 µL 2 × Rapid Taq Master Mix (Vazyme, Nanjing, China), 8 µL ddH_2_O, 1 µL cDNA, and 1 µL each of the forward and reverse amplification primers, resulting in a final volume of 20 µL. To perform qRT-PCR, LightCycler^®^ 480 software (Roche, Shanghai, China) was used. All samples were subjected to three technical replicates and three biological replicates. The reaction mixture comprised 10 µL 2 × Taq Pro Universal SYBR qPCR Master Mix (Vazyme, Nanjing, China), 7.2 µL ddH_2_O, 2 µL cDNA, and 0.4 µL each of the forward and reverse amplification primers for a final volume of 20 µL. The cDNAs diluted several times were used as qRT-qPCR amplification templates. A curve was drawn to obtain the R^2^ value, and the amplification efficiency was calculated according to the efficiency calculation formula.

### 2.5. Stability Analysis of Candidate Reference Genes

The sample cycle threshold (Ct) value was obtained according to the RT-qPCR experiment. The data were processed using GeNorm (https://genorm.cmgg.be/, accessed on 24 August 2023), NormFinder (https://genorm.cmgg.be/, accessed on 24 August 2023), and BestKeeper (https://www.gene-quantification.de/bestkeeper.html, accessed on 24 August 2023) to obtain the order of the stability of the internal reference genes. RefFinder (http://blooge.cn/RefFinder/, accessed on 24 August 2023) was used to comprehensively evaluate the analysis results of the above three programs to obtain stable reference genes. The stable reference genes were selected to analyze the expression patterns of the *Vradi0146s00260*, *Vradi0158s00480*, *Vradi07g23860*, and *Vradi11g03350* genes under infection by *P. myriotylum*. According to the expression trend and RNA-seq trend analysis, the selected reference genes were further verified.

## 3. Results

### 3.1. Primer Specificity and Amplification Efficiency of Candidate Reference Genes

**Figure 1:**
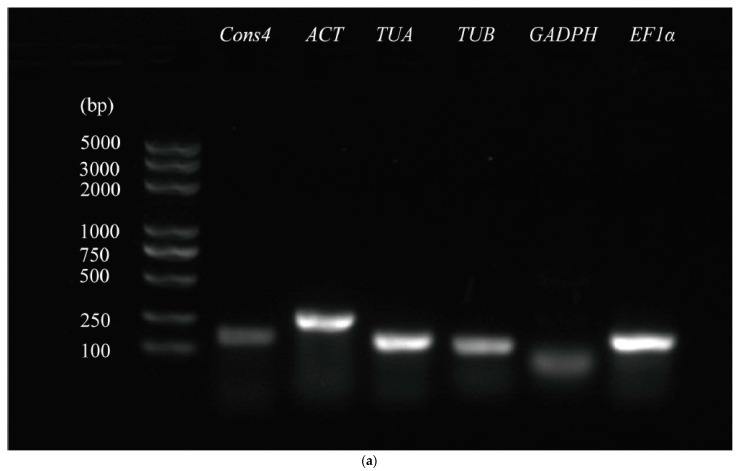

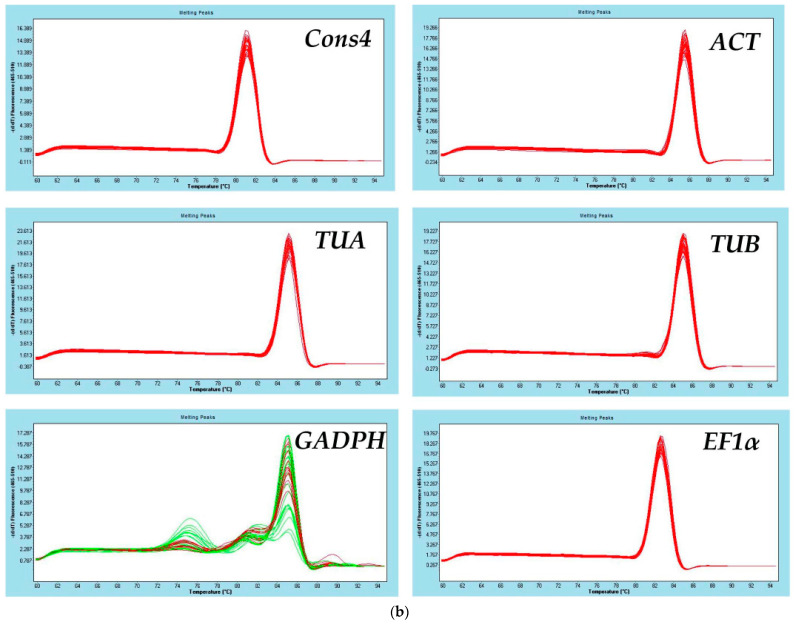
Primer specificity of candidate reference genes. (**a**) The primer specificity of candidate reference genes was detected by agarose electrophoresis; (**b**) melting curve of qRT-PCR for candidate internal reference genes in mung bean.

The PCR amplification products of the six candidate reference genes (*Cons4*, *ACT*, *TUA*, *TUB*, *GAPDH*, and *EF1α*) were analyzed via 1% agarose gel electrophoresis using the cDNA of each sample as a template. The amplified band size was consistent with the expected fragment sizes, and the band was single without a primer dimer, as shown in Figure 1a. After qRT-qPCR detection, the melting curve of each reference gene primer was obtained, as depicted in Figure 1b. The *GAPDH* reference gene showed a double signal peak, while the other five reference genes showed a single signal peak. The primer amplification efficiency was ideal, and the amplification specificity was high.

### 3.2. Expression Analysis of Candidate Reference Genes under Biotic Stress and Hormone Treatment

**Figure 2:**
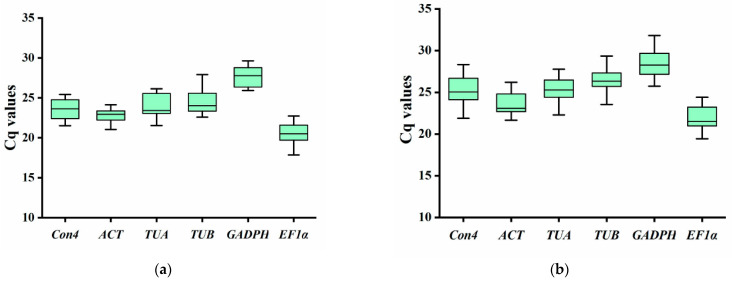
Ct value ranking of candidate internal reference genes (**a**) in hormone-treated samples and (**b**) in biotic stress samples. The boxes indicate the 25th and 75th percentiles. Error bars represent maximum and minimum values. The line across each box indicates the median.

Three biological replicates were set up for the roots of mung bean treated with four soil-borne disease pathogens and five hormones at different time points. The higher the Ct value, the smaller the template concentration and the lower the gene expression. The change in Ct value between different treatments reflects the difference in gene expression between samples. In the samples from different treatments, the Ct value distribution of the six candidate reference genes was different. Under the condition of hormone treatment, the Ct value ranged from a minimum of 17.87 for *EF1α* to a maximum of 29.63 for *GAPDH*. The highest average expression among the candidate reference genes was found for *EF1α*, with an average Ct value of 20.74, and the lowest average expression was found for *GAPDH*, with an average Ct value of 27.67. In addition, the Ct value of *TUB* showed the largest difference, ranging from 22.60 to 27.91, with a difference of 5.30 (Figure 2a). Under the condition of pathogen infection, the Ct value ranged from a minimum of 19.43 for *EF1α* to a maximum of 31.82 for *GAPDH*. The highest average expression of candidate reference genes was found for *EF1α*, with an average Ct value of 22.03. The lowest average expression was found for *GAPDH*, with an average Ct value of 28.44. In addition, the Ct value of *Cons4* was the largest, ranging from 21.89 to 28.32, with a difference of 6.43 (Figure 2b). The expression of these reference genes is unstable. Under certain conditions, appropriate reference genes must be screened.

### 3.3. Stability Analysis of the Candidate Reference Genes

**Figure 3:**
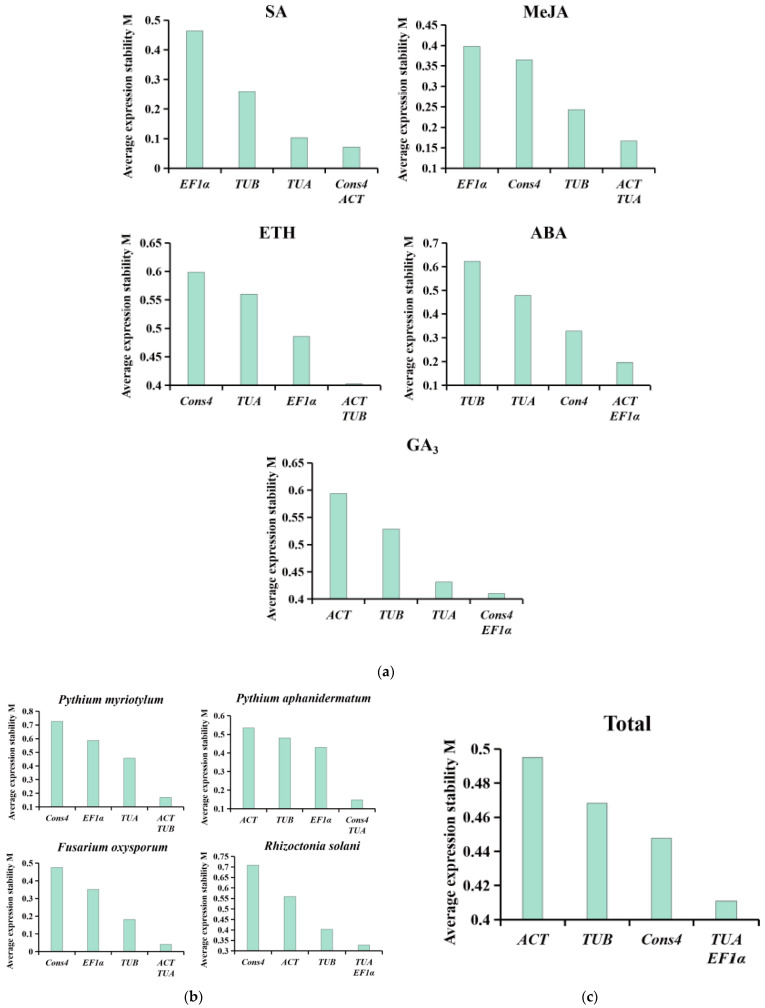
The average expression stability values (M) of the candidate internal reference genes were analyzed and ranked by GeNorm calculation. (**a**) Five hormone treatments (100 μM SA, 50 μM MeJA, 1 mM ETH, 50 μM ABA, 50 μM GA_3_); (**b**) four soil-borne disease pathogen treatments (*P. myriotylum*, *P. aphanidermatum*, *F. oxysporum*, and *R. solani*); (**c**) overall analysis of the results of the nine treatments. A lower average expression stability (M value) indicates more stable expression.

The stability value M was determined using the statistical software GeNorm, and the M values obtained under different treatments for the five internal reference genes were sorted. The smaller the M value, the more stable the expression of the internal reference gene. The M values of the five internal reference genes in this study were all available (M < 1.5). The M value of *Cons4* and *ACT* under SA treatment was the lowest (M = 0.071), and their expression was the most stable. We found that under MeJA treatment, the expression of the *ACT* and *TUA* genes had the lowest M value (M = 0.167). Meanwhile, under ETH treatment, the expression of the *ACT* and *TUB* genes was the most stable (M = 0.403). When subjected to ABA and GA_3_ treatments, the expression of the *ACT* and *EF1α* genes (M = 0.197), and that of the *Cons4* and *EF1α* genes (M = 0.410), was found to be the most stable. In terms of pathogen infection, the most stably expressed genes were *ACT* and *TUB* (M = 0.170) in *P. myriotylum* infection, *Cons4* and *TUA* (M = 0.147) in *P. aphanidermatum* infection, *ACT* and *TUA* (M = 0.041) in *F. oxysporum* infection, and *TUA* and *EF1α* (M = 0.328) in *R. solani* infection. Overall, the analysis showed that the *TUA* and *EF1α* genes had the best stability (M = 0.411) (Figure 3).

**Figure 4:**
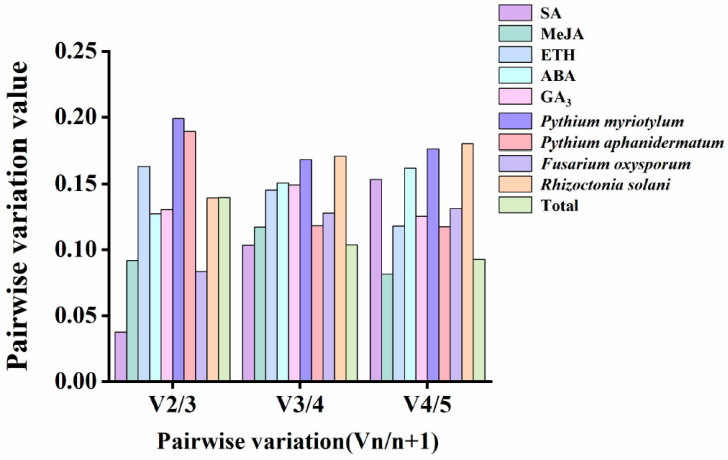
Pairwise variation (V) of candidate internal reference genes.

GeNorm software can use multiple internal reference genes to analyze pairwise differences and obtain an optimal pairwise variation value (Vn/n + 1). This is the criterion for selecting the optimal number of internal reference genes. If Vn/n + 1 is less than the software default limit of 0.15, the optimal number of internal reference genes is n + 1. An internal reference gene is not required to remove differences. If it exceeds 0.15, the number of internal reference genes should be increased. Treatments with SA, MeJA, ABA, GA_3_, *F. oxysporum*, and *R. solani*, and a total V2/V3 of <0.15 indicate that two internal reference genes are required for proper comparison of the target gene expression data. For ETH and *P. aphanidermatum* treatments, V3/V4 < 0.15, so three internal reference genes are required. In the *P. myriotylum* treatment, V4/V5 was greater than 0.15, requiring a fifth internal reference gene to eliminate differences (Figure 4).

The NormFinder add-on for Microsoft Excel helps one choose the best gene to use as reference by calculating a stability value for how much the gene is expressed. The lower the stability value, the more stable the expression of the reference gene. We found that different genes acted as the most stable reference gene under different treatments. Specifically, under the SA and GA_3_ treatments, the most stable reference gene was *Cons4*, with stability values of 0.025 and 0.070, respectively. MeJA, ETH, and *P. myriotylum* treatments showed *ACT* to be the most stable reference gene, with stability values of 0.109, 0.122, and 0.202, respectively. *EF1α* was found to be the most stabile reference gene under ABA and *P. aphanidermatum* treatment and overall level analysis, with stability values of 0.161, 0.089, and 0.124, respectively. *TUA* was identified as the most stable reference gene under *R. solani* treatment, with a stability value of 0.114. *TUB* was determined to be the most stable reference gene under *F. oxysporum* treatment, with a stability value of 0.077 (Table 2 and Table 3).

We further analyzed the expression stability of the candidate internal reference genes by obtaining the standard deviation (SD) and coefficient of variation (CV) through BestKeeper analysis of the Ct value of the internal reference genes. The software identified *TUB* as the most stable reference gene under SA and MeJA treatments, while *Cons4* was the most stable under ETH, ABA, and GA_3_ treatments (Table 4). Under treatment with *P. myriotylum*, *P. aphanidermatum*, *F. oxysporum*, and *R. solani*, *TUB*, *ACT*, *TUA*, and *TUB*, respectively, were the most stable reference genes. Overall, *TUA* was found to be the most stable reference gene, as indicated in Table 5.

The stability of the five candidate reference genes was analyzed using GeNorm, NormFinder, and BestKeeper, and the results were different. Therefore, the results regarding the most stable reference genes analyzed by the three software products were comprehensively evaluated using RefFinder. In the overall level analysis, the most stable internal reference gene was *TUA*. Under SA, ABA, GA_3_, and *P. myriotylum* treatment, the most stable reference gene was *TUA*. Under ETH treatment, the most stable reference gene was *TUB*. The most stable reference gene under MeJA and *F. oxysporum* treatment was *ACT*. *EF1α* was the most stable reference gene under *P. aphanidermatum* and *R. solani* treatment (Table 6).

### 3.4. Stability Analysis of the Candidate Reference Genes

**Figure 5:**
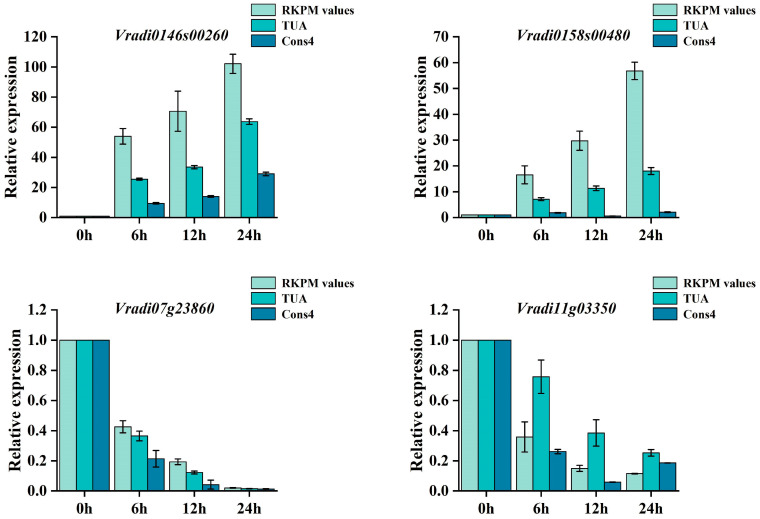
Relative expression of genes at different time points after treatments of *P myriotylum* normalized to the expression of the most stable reference gene (*TUA*) and least stable reference gene (*Cons4*). The error bar indicates the standard error.

According to the results of the RefFinder comprehensive evaluation, *TUA* was selected as the most stable reference gene and *Cons4* was selected as the least stable reference gene. To further verify the reliability of the results, qRT-PCR analysis was performed on four selected genes (*Vradi0146s00260*, *Vradi0158s00480*, *Vradi07g23860*, and *Vradi11g03350*) with *TUA* and *Cons4* as the internal reference genes. When the most stable reference gene (*TUA*) was selected, the expression levels of *Vradi0146s00260*, *Vradi0158s00480*, *Vradi07g23860*, and *Vradi11g03350* at 6 hpi, 12 hpi, and 24 hpi showed a higher similarity to the fold change observed in the RNA-seq data. Meanwhile, the qRT-PCR trend for the four target genes was consistent with the RNA-seq data analysis results. However, the target genes’ expression levels varied greatly in the case of *Cons4* (Figure 5). In conclusion, the results of the stability analysis were reliable.

## 4. Discussion

qRT-PCR is a powerful tool for quantitative analysis of gene expression [37]. The data obtained by qRT-PCR must be corrected using internal reference genes to increase their reliability. However, the expression of commonly used reference genes varies under different conditions in different species, which requires us to select stable reference genes according to the corresponding experimental materials and experimental conditions [38]. In this study, five candidate reference genes were found to be highly specific (Figure 1), and an expression analysis was conducted in root tissues after various biotic stresses and hormone treatments.

To assess the stability of the reference genes, we utilized four different statistical methods. The results from GeNorm and NormFinder showed similar stability rankings for the reference genes [39,40,41], while BestKeeper analysis generated different rankings. Specifically, GeNorm and NormFinder identified *ACT* as the most stable reference gene under MeJA, ETH, and *P. myriotylum* treatments (Figure 4, Table 2). However, the BestKeeper results showed that *TUB* and *Cons4* had the most stable expression under the MeJA, ETH, and *P. myriotylum* treatments (Table 3). RefFinder was further used to comprehensively evaluate the ranking results of the first three software products. The findings revealed that *TUA* exhibited the highest stability in the overall level analysis and in SA, ABA, GA_3_, and *P. myriotylum* treatment. *TUA* has a number of important functions. It not only is an important component of the cytoskeleton but also participates in various life activities, such as intracellular material transportation and shape maintenance. Previous studies have shown that *TUA* can serve as a suitable reference gene for various stages of female flower bud differentiation in *Juglans regia* [42], somatic embryogenesis (SE) in *Liriodendron chinense* [43], and abiotic stress in *Diospyros kaki Thunb* [44]. Our results also showed that *ACT* can be used as a reference gene in the root of mung bean under MeJA treatments, and it has been shown to serve as a reference gene in *Vigna angularis* under biotic stresses and as a reference gene in *Glycyrrhiza* adapted to abiotic stresses [45]. However, in *Cucurbitaceae*, *TUA* was found to be the most unstable reference gene under oxidative stresses and plant growth regulators [46].At the same time, *ACT* is not suitable as an internal reference gene under biotic stress in maize and sunflower [29]. Therefore, it is evident that there is no ideal reference gene that can be used for all experiments. To verify the reliability of the selected candidate reference genes, we selected four genes that responded severely after *P. myriotylum* infection based on transcriptome data. Among them, *Vradi0146s00260* and *Vradi0158s00480* are members of the WRKY family, and their expression was upregulated during *P. myriotylum* infection. On the other hand, *Vradi07g23860* and *Vradi11g03350* belong to the JAZ family, and their expression was downregulated during *P. myriotylum* infection. According to previous research, WRKY transcription factors can regulate a variety of plant biological process signal networks such as salicylic acid (SA), ethylene (ET), and jasmonic acid (JA) [47,48]. The jasmonate ZIM-domain (JAZ) protein is an important negative regulator in the JA signaling pathway [49,50]. WRKY and JAZ play an important role in plant defense response. *MdWRKY75e* overexpression strengthens the resistance to *Alternaria alternata*, mainly via the jasmonic acid (JA) signaling pathway [48]. Additionally, *SlWRKY30* decreases the severity of *Ralstonia solanacearum* infection (RSI)-induced bacterial wilt in tomato, increased H_2_O_2_ accumulation and cell necrosis, and it was a positive regulator of RSI in tomato [51]. Silencing of *JAZ1/2* en-hances soybean resistance against *Phytophthora sojae* [50]. We further used the most stable reference gene (*TUA*) and the least stable reference gene (*Cons4*) to standardize the relative expression levels of the *Vradi0146s00260*, *Vradi0158s00480*, *Vradi07g23860*, and *Vradi11g03350* genes under *P. myriotylum* infection. The results showed that the expression levels of the target genes were in general agreement with the RNA-seq data when we selected *TUA* as the reference gene.

## 5. Conclusions

Our study showed that the expression stability varied among the five candidate reference genes under various biotic stresses and hormone treatments. An analysis using four programs (GeNorm, NormFinder, BestKeeper, and RefFinder) showed that *TUA* was the best reference gene for qRT-PCR analysis. *ACT* was the most stable reference gene under MeJA and *F. oxysporum* treatment. *EF1α* was identified as the most stable reference gene under *P. aphanidermatum* and *R. solani* treatments. The findings of this study can be used as a valuable reference for selecting appropriate internal reference genes when studying resistance genes under mung bean root rot infection conditions. Furthermore, this study provides a strong basis for future research on the role of mung bean root rot resistance-related genes in the defense response against *P. myriotylum*.

## Figures and Tables

**Table 1:** Information on the candidate reference genes for mung bean and their amplicon characteristics.

Gene Name	Gene ID	Primer Sequence F/R (5′-3′)	Amplicon Length (bp)	Tm (°C)	Efficiency (%)	R^2^
*Cons4*	Vradi11g01010	F:TCGCCAGATATTGCAGATAA	156	81.24	104.43	0.996
		R:GGAGGAACAGTAGAAAGGGT				
*ACT*	Vradi03g00210	F:GGCGGTGTTCCCTAGCATTG	246	85.59	95.96	0.999
		R:AGCGGTGCCTCGGTAAGAAG				
*TUA*	Vradi08g19840	F:GGTCAAATGCCAAGTGACAAAACAG	150	85.21	97.392	0.998
		R:GTAAGGTCCAGTCCTAACCTCATCG				
*TUB*	Vradi05g13910	F:GCTTATGGATCTTGAACCTGGAA	136	85.11	97.869	0.997
		R:GCCTTCGGTATAATGACCTTTCG				
*GADPH*	Vradi0043s00410	F:CGTTTTCACCCCTTTTCCG	258	85.06	113.781	0.983
		R:CGTGATGCTTCCAGTGTCCG				
*EF1α*	Vradi01g08330	F:AGCGTGAAAGAGGAATTACCATCG	153	82.65	98.095	0.999
		R:CAATAATAAGGACAGCACAATCAG				

**Table 2:** Analysis and ranking, using NormFinder, of the expression stability values of candidate internal reference genes under five hormone treatments.

Gene Name	SA		MeJA		ETH		ABA		GA_3_	
	Stability Value	Rank	Stability Value	Rank	Stability Value	Rank	Stability Value	Rank	Stability Value	Rank
*Cons4*	0.024676533	1	0.222221876	3	0.384529941	5	0.259344484	3	0.069960504	1
*ACT*	0.024676533	2	0.109242042	1	0.122377254	1	0.333505004	4	0.413025931	5
*TUA*	0.037894549	3	0.147671416	2	0.339757665	3	0.211394671	2	0.176462268	2
*TUB*	0.377236807	4	0.253280028	4	0.287658317	2	0.547498395	5	0.379257235	4
*EF1α*	0.525706883	5	0.266094327	5	0.370284053	4	0.160752498	1	0.332945438	3

**Table 3:** Analysis and ranking, using NormFinder, of the expression stability value and the overall level stability values of candidate internal reference genes under four soil-borne disease pathogen treatments.

Gene Name	*Pythium myriotylum*		*Pythium aphanidermatum*		*Fusarium oxysporum*		*Rhizoctonia solani*		Total	
	Stability Value	Rank	Stability Value	Rank	Stability Value	Rank	Stability Value	Rank	Stability Value	Rank
*Cons4*	0.590638821	5	0.309761038	3	0.444615042	5	0.60680042	5	0.231731888	4
*ACT*	0.202036918	1	0.387614988	5	0.251700828	4	0.530437025	4	0.299557287	5
*TUA*	0.445402607	4	0.337951776	4	0.235601773	3	0.113789254	1	0.247803221	3
*TUB*	0.274850505	3	0.184158256	2	0.076710811	1	0.316568757	3	0.285054343	2
*EF1α*	0.253857199	2	0.088874014	1	0.168786015	2	0.113789254	2	0.124083336	1

**Table 4:** Analysis of the expression stability of candidate reference genes under five hormone treatments according to BestKeeper. CV: coefficient of variation; SD: standard deviation.

Gene Name	SA			MeJA			ETH			ABA			GA_3_		
	CV	SD	Rank	CV	SD	Rank	CV	SD	Rank	CV	SD	Rank	CV	SD	Rank
*Cons4*	3.7	0.86	4	3.47	0.79	5	3.07	0.73	1	4.5	1.07	1	4.92	1.15	1
*ACT*	3.74	0.83	3	1.95	0.42	2	4.5	1.02	2	4.99	1.13	2	3.67	0.82	2
*TUA*	3.38	0.77	2	2.52	0.56	3	5.32	1.26	4	6.49	1.55	3	5.73	1.35	3
*TUB*	2.04	0.48	1	1.37	0.31	1	4.74	1.17	3	7.36	1.85	5	5.06	1.21	5
*EF1α*	6.37	1.26	5	4.09	0.78	4	7.71	1.55	5	8.18	1.67	4	7.27	1.45	4

**Table 5:** Analysis of the expression stability and the overall level stability of candidate internal reference genes under four soil-borne disease pathogen treatments, according to BestKeeper. CV: coefficient of variation; SD: standard deviation.

Gene Name	*Pythium myriotylum*			*Pythium aphanidermatum*			*Fusarium oxysporum*			*Rhizoctonia solani*			Total		
	CV	SD	Rank	CV	SD	Rank	CV	SD	Rank	CV	SD	Rank	CV	SD	Rank
*Cons4*	5.38	1.35	5	4.64	1.09	4	4.55	1.2	5	5.53	1.45	4	5.89	1.44	5
*ACT*	2.45	0.56	2	2.67	0.6	1	2.52	0.62	2	6.58	1.66	5	4.48	1.04	1
*TUA*	3.49	0.86	3	5.02	1.21	5	2.3	0.6	1	4.55	1.2	3	5.72	1.41	3
*TUB*	1.72	0.45	1	3.86	0.97	3	2.44	0.65	3	3.25	0.92	1	5.55	1.42	4
*EF1α*	4.73	1.04	4	3.56	0.73	2	4.22	0.97	4	4.94	1.12	2	5.7	1.22	2

**Table 6:** Comprehensive ranking of candidate reference gene stability analyzed using RefFinder.

Rank	Hormone Induction					Biotic Stress				
	SA	MeJA	ETH	ABA	GA_3_	*Pythium myriotylum*	*Pythium aphanidermatum*	*Fusarium oxysporum*	*Rhizoctonia solani*	Total
1	*TUA*	*ACT*	*TUB*	*TUA*	*TUA*	*TUA*	*EF1α*	*ACT*	*EF1α*	*TUA*
2	*TUB*	*TUB*	*ACT*	*EF1α*	*TUB*	*EF1α*	*Cons4*	*TUA*	*TUA*	*EF1α*
3	*ACT*	*TUA*	*EF1α*	*TUB*	*ACT*	*TUB*	*TUB*	*EF1α*	*TUB*	*ACT*
4	*EF1α*	*EF1α*	*TUA*	*Cons4*	*EF1α*	*ACT*	*TUA*	*Cons4*	*Cons4*	*TUB*
5	*Cons4*	*Cons4*	*Cons4*	*ACT*	*Cons4*	*Cons4*	*ACT*	*TUB*	*ACT*	*Cons4*

## Data Availability

Not applicable.
